# Determination of aortic pulse transit time based on waveform decomposition of radial pressure wave

**DOI:** 10.1038/s41598-021-99723-w

**Published:** 2021-10-11

**Authors:** Wenyan Liu, Daiyuan Song, Yang Yao, Lin Qi, Liling Hao, Jun Yang, Hongxia Ning, Lisheng Xu

**Affiliations:** 1 College of Medicine and Biological Information Engineering, Northeastern University, Shenyang, 110167 China; 2 The First Hospital of China Medical University, Shenyang, 110122 China; 3 Key Laboratory of Medical Image Computing, Ministry of Education, Shenyang, 110169 China; 4 Neusoft Research of Intelligent Healthcare Technology, Co. Ltd., Shenyang, 110169 China

**Keywords:** Biomedical engineering, Arterial stiffening, Predictive markers, Risk factors

## Abstract

Carotid-femoral pulse transit time (cfPTT) is a widely accepted measure of central arterial stiffness. The cfPTT is commonly calculated from two synchronized pressure waves. However, measurement of synchronized pressure waves is technically challenging. In this paper, a method of decomposing the radial pressure wave is proposed for estimating cfPTT. From the radial pressure wave alone, the pressure wave can be decomposed into forward and backward waves by fitting a double triangular flow wave. The first zero point of the second derivative of the radial pressure wave and the peak of the dicrotic segment of radial pressure wave are used as the peaks of the fitted double triangular flow wave. The correlation coefficient between the measured wave and the estimated forward and backward waves based on the decomposition of the radial pressure wave was 0.98 and 0.75, respectively. Then from the backward wave, cfPTT can be estimated. Because it has been verified that the time lag estimation based on of backward wave has strong correlation with the measured cfPTT. The corresponding regression function between the time lag estimation of backward wave and measured cfPTT is y = 0.96x + 5.50 (r = 0.77; p < 0.001). The estimated cfPTT using radial pressure wave decomposition based on the proposed double triangular flow wave is more accurate and convenient than the decomposition of the aortic pressure wave based on the triangular flow wave. The significance of this study is that arterial stiffness can be directly estimated from a noninvasively measured radial pressure wave.

## Introduction

Increased conduit artery stiffness is associated with ageing and many types of arterial disease and carotid-femoral aortic pulse wave velocity (PWV) is a widely accepted assessing arterial stiffness^1^. Carotid-femoral pulse wave velocity (cfPWV) can be calculated from two synchronized pressure waves obtained noninvasively and is a reliable surrogate for aortic PWV^2^. The cfPWV is generally calculated by the pulse transit time (PTT) from carotid artery to femoral artery^3–5^. However, there remain some barriers to its widespread adoption as a routine clinical procedure, especially in the measurements of pressure wave and the true distance between carotid and femoral measurement sites^6,7^. In practice, the carotid and femoral pressure waves are recorded simultaneously or determined with reference to the R wave of the ECG signal. The femoral pressure wave cannot be readily obtained when the patients are obese. Therefore, it is necessary to explore a simplified method to calculate the cfPTT^8–10^.

Pressure waves are formed by the contractions of the heart and are transmitted and reflected in the arterial system^11^. Wave reflection is a very important concept in arterial pressure wave analysis^12–14^. Wave reflection index, which is defined as the ratio of the forward wave and backward (reflected) wave, is related to arterial stiffness^15–17^. Westerhof’s study^17^ also showed that the forward and backward pressure waves can be extracted from the measured pressure wave and the triangular flow wave fitting method^18^. The first zero point of the second derivative of the aortic pressure wave can be used to represent the beginning of the reflected wave. Then forward and backward waves can be determined from the triangular flow wave and measured pressure wave^19,20^. Qasem et al. improved the method by introducing a regression model to estimate cfPTT from time difference between the forward wave and backward wave in human aorta^21^. This time difference (TR_2_) can be calculated from the cross-correlation between the two waves. The calculated TR_2_ is strongly correlated with the cfPTT, so the cfPWV can be estimated from the distance of carotid artery and femoral artery divided by TR_2_/2, where the factor 2 allows for the combined distance travelled by the forward-going and reflected pressure waves. Studies have shown that the reflection wave becomes progressively greater with age, and returns progressively earlier. The age-dependent decomposition of radial pressure wave has been proposed by Gwanghyun et al.^22^. The decomposition model contains two triangular components neither of which is derived from the measured pressure wave. However, the estimation of arterial stiffness still has some problems due to the difficulty of acquiring the aortic pressure wave noninvasively. Although there is a variety of ways to estimate this wave, such as surrogate methods, transfer function (TF) methods, blind system identification (BSI) methods and so on, the reconstruction of the aortic pressure wave still has many drawbacks, so that useful clinical information contained in the ‘true’ aortic pressure wave may not be directly derivable from radial pressure wave. Hence, there will be mistakes in the recognition of the feature points of aortic pressure wave after reconstruction, which may result in some errors in the decomposition^23–25^.

In this study, a novel method is proposed to estimate the cfPTT from radial pressure wave alone. The method aims to fit the flow wave of the radial artery using two triangles, one to simulate systole and the other to simulate diastole. Then the forward and reflected waves can be derived from the measured radial pressure wave and the fitted double triangular radial flow wave. The decomposition of the radial pressure wave is evaluated by the measured radial pressure wave and flow wave recordings. cfPTT can be calculated from the time difference of the radial backward wave. The estimated PTT is compared to the measured cfPTT and that from previous studies. A regression model between relating the estimated aortic PTT and measured cfPTT is obtained from 116 recordings of carotid and femoral pressure waves. Using a database of virtual healthy subjects, the relation between the radial and aortic backward waves is verified.

## Materials and methods

### Data acquisition

Ultrasonic equipment (Smart V-Link) and SphygmoCor device (SphygmoCor, AtCor Medical, Sydney, Australia) were used. The blood flow wave was extracted from the envelope of ultrasound data. Pressure wave recordings from the SphygmoCor device were sampled at 128 Hz. For the validation of double triangular flow wave, 35 sets of recordings were used including radial pressure and radial blood flow waves. The detailed follow-up records are listed in dataset 1 of Table 1. Dataset 1 includes the measured radial pressure wave and measured radial flow wave.

**Table 1:** Demographics of the volunteers for the dataset of 35 and 116, data are presented in mean ± SD.

	Dataset 1	Dataset 2
Total (n)	35	116
Males (n)	22	64
Females (n)	13	52
Age (yr.)	31 ± 17	43 ± 21
Height (cm)	171 ± 10	168 ± 8
Weight (kg)	63 ± 15	65 ± 11
Systolic BP (mmHg)	116 ± 13	119 ± 15
Diastolic BP (mmHg)	69 ± 11	74 ± 10
Heart rate (bmp)	71 ± 10	68 ± 10

In addition, there were 116 individuals with pressure wave and cfPTT. The ratio of training set to test set was 8:2. The radial pressure wave was measured noninvasively and aortic pressure wave calculated using the SphygmoCor device with a generalized transfer function. The cfPTT was calculated from the foot-to-foot time delay. Brachial systolic and diastolic pressure values were measured using a brachial cuff oscillometric sphygmomanometer. Immediately after the brachial blood pressure measurement, carotid and femoral pressure waves were simultaneously measured two times by applanation tonometry using two transducers one at the right common carotid artery and the other at the femoral artery, at a sampling rate of 1 kHz. Acquisition equipment is from Techman of Chengdu, China. The direct distance was measured with a steel tape measure between the carotid and femoral arterial sites. 80% of the direct distance between the common carotid and femoral arteries has the smallest error and the highest correlation^26^. In this study, the pulse traveled distance is estimated by the 80% of the direct distance. It is known as the effective reflecting distance. The detailed follow-up records are shown in dataset 2 of Table 1. Approval was obtained from the Research Ethics Committee of the Northeastern University (EC-2020B017), and written informed consent was obtained from all participants. All authors confirm that the research is performed in accordance with relevant guidelines and regulations. Every volunteer was in a supine position for data collection. The pulse wave and flow wave were collected at the same part of radial artery in same location, so they cannot be synchronized, but not more than 5 min. A total of 151 volunteers took part in the data acquisition, of which 35 provided the radial flow wave and pressure wave, and 116, the radial arterial pressure wave and cfPTT. The volunteers were all healthy adults.

The database of virtual healthy subjects’ pressure wave was used to observe the influence of arterial parameters on the backward wave. The database uses a validated one-dimension model with parameters varied within the healthy range to generate 3325 sets of pressure and flow waves of virtual healthy adult subjects. There are seven parameters including elastic arterial PWV (*V*_*e*_), muscular arterial PWV (*V*_*m*_), elastic arterial diameter (*D*_*e*_), muscular arterial diameter (*D*_*m*_), heart rate (HR), stroke volume (SV), and peripheral vascular resistance (R) of 55 arteries, varying in different levels, which cause a change in the wave^27^.

### Wave decomposition analysis

**Figure 1:**
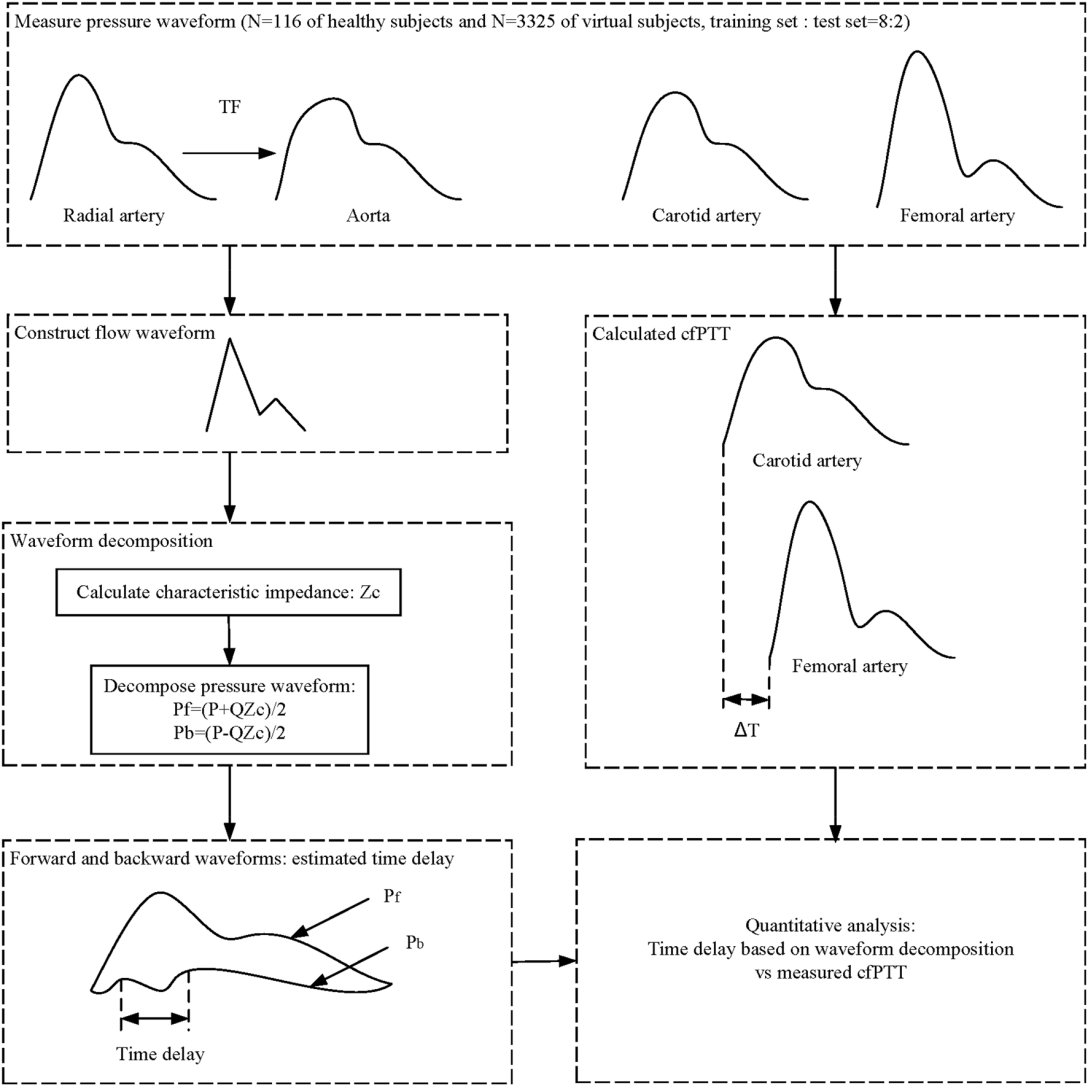
The block diagram of the time delay estimation methodology.

**Figure 2:**
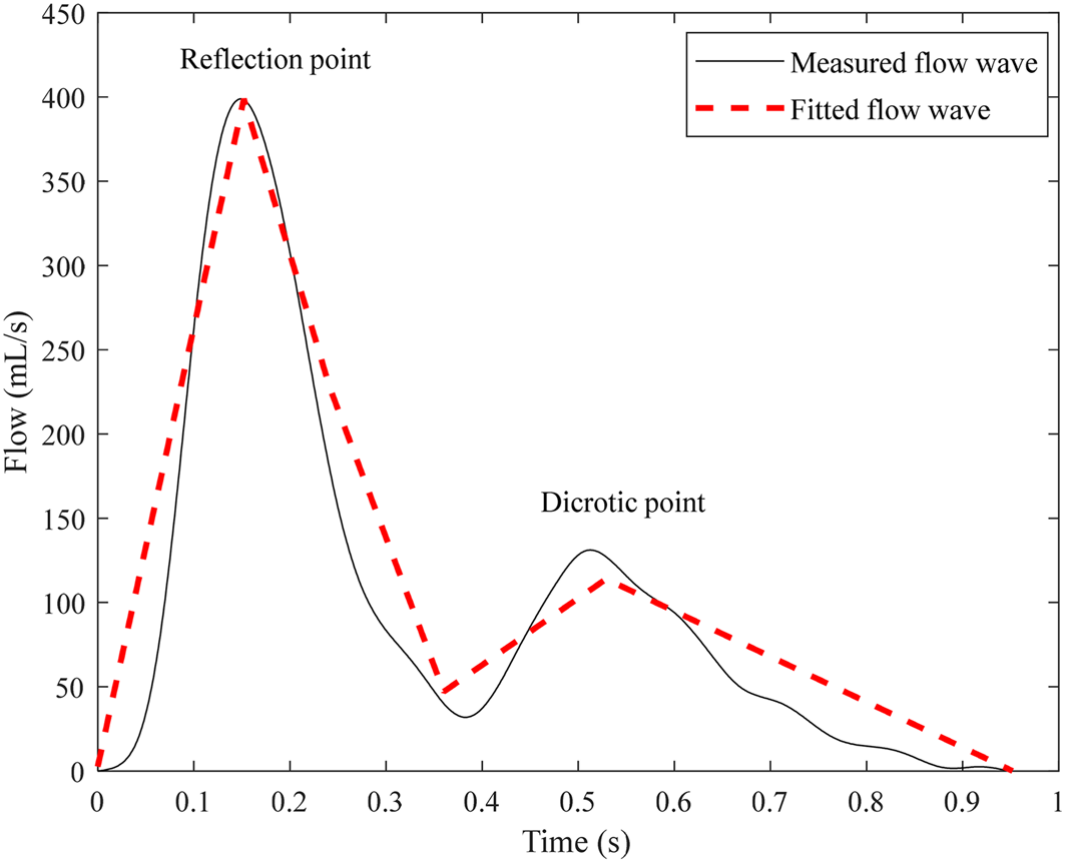
Calculated and measured blood flow waves.

As shown in the Fig. 1, the block diagram of the time delay estimation methodology is given. A double triangular wave can be fitted to estimate the flow wave from the radial pressure wave, as shown in Fig. 2. Then the fitted double triangular wave is used to replace the flow wave Q_m_(*t*) in Eqs. (3) and (4). *Z*_*c*_ is the characteristic impedance of the corresponding artery, and can be calculated from frequency or time domains^28^. Based on previous research, calibration of the flow wave is not required^17^. The pressure wave, P_m_(*t*) can be directly measured from the radial artery noninvasively. Both blood flow and pressure waves can be regarded as a superposition of forward and backward waves^29,30^. The effect of reflections on the pressure wave and flow wave is opposite. The measured waves, P_m_(*t*) and Q_m_(*t*) can be expressed as follows:

1 $$ {\text{P}}_{{\text{m}}} (t) = {\text{P}}_{{\text{f}}} (t) + {\text{P}}_{{\text{b}}} (t) $$

2 $$ {\text{Q}}_{{\text{m}}} (t) = {\text{Q}}_{{\text{f}}} (t) - {\text{Q}}_{{\text{b}}} (t) $$

P_m_(*t*) and Q_m_(*t*) represent the measured pressure wave and flow wave, respectively. P_f_(*t*) and Q_f_(*t*) represent the forward wave, P_b_(*t*) and Q_b_(*t*) represent the backward wave. Then the forward wave, P_f_(*t*) and backward wave, P_b_(*t*) can be calculated from P_m_(*t*) and Q_m_(*t*) with transmission line analysis as follows:

3 $$ {\text{P}}_{{\text{f}}} (t) = [{\text{P}}_{{\text{m}}} (t) + {\text{Z}}_{{\text{c}}}  \times {\text{Q}}_{{\text{m}}} (t)]/{\text{2}} $$

4 $$ {\text{P}}_{{\text{b}}} (t) = [{\text{P}}_{{\text{m}}} (t) - {\text{Z}}_{{\text{c}}}  \times {\text{Q}}_{{\text{m}}} (t)]/{\text{2}} $$

The two peaks of triangle shape occur at the time of the first systolic inflection (reflection point) and peak of the dicrotic segment, respectively^20^. The arterial input impedance was calculated in the frequency domain, and the characteristic impedance was derived from the averaged value of the 4th to 7th harmonic of the input impedance modulus. Two triangular waves were used to fit the systolic and diastolic blood flow waves. The timing of the first triangle is determined based on the ejection duration and the second triangle is based on the duration of diastole. In order to fit the blood flow wave accurately, the height of flow wave at the end of systole was calculated. The two triangles need to overlap a little at the intersection, to make the double triangular wave more similar to measured flow wave. The base of the second triangle intersects the first triangle by a tenth of the heart interval, which has achieved a better decomposition. The degree of overlap was generalized from all the collected flow waves. The reflection point is obtained from the first zero point of the second derivative of the pulse wave.

**Figure 3:**
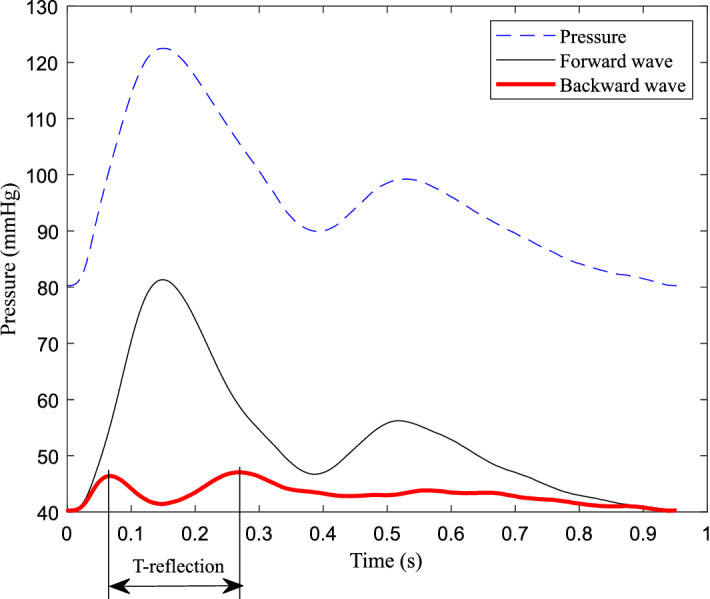
Decomposition of the pulse wave in the radial artery.

The radial pulse wave is decomposed into forward and backward waves through the double triangular flow wave, as shown in Fig. 3. From the backward wave, the time lag can be calculated between the fluctuations. Using the measured radial flow wave, the forward wave P_f_(*t*) and backward wave P_b_(*t*) can be calculated through Eqs. (3) and (4). The results of decomposition from measured radial flow wave were used as the ‘ground truth’, which was used to compare with the estimated decomposition based on the double triangular method. RMSE errors and correlation of the forward and backward waves were calculated. All feature points of the double triangular flow wave were determined according to the relationship between the radial pressure and blood flow waves. The selection of feature points was evaluated by calculating the correlation between the measured radial flow and double triangular flow waves. A record of the radial pressure wave during 5 min was decomposed for testing the robustness of the double triangular model. The elasticity of the radial artery can be assessed from the decomposition directly^31^. Reflection magnitude (RM) and reflection index (RI) were calculated from Eqs. (5) and (6).

5 $$ {\text{RM }} = {\text{ }}\left| {{\text{P}}_{{\text{b}}} } \right|/\left| {{\text{P}}_{{\text{f}}} } \right| $$

6 $$ {\text{RI }} = {\text{ }}\left| {{\text{P}}_{{\text{b}}} } \right|/\left( {\left| {{\text{P}}_{{\text{f}}} } \right|{\text{ }} + {\text{ }}\left| {{\text{P}}_{{\text{b}}} } \right|} \right) $$

**Figure 4:**
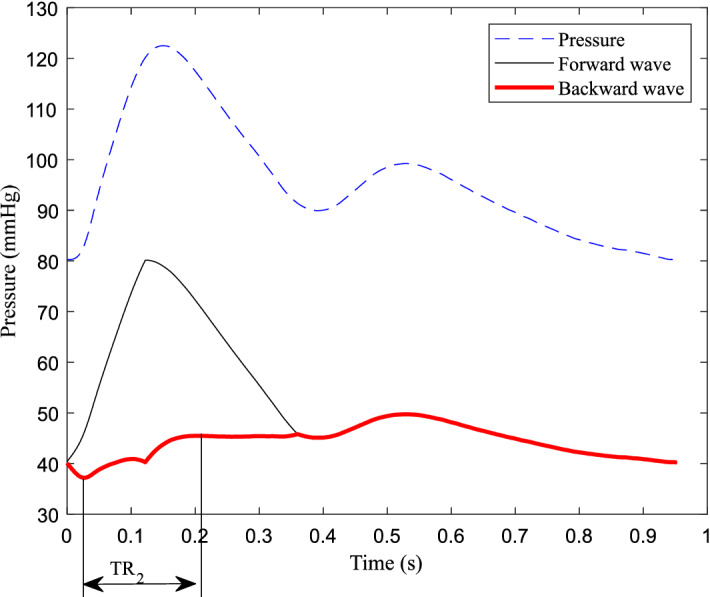
The time difference between forward wave and backward wave of aortic pressure wave.

The fluctuation of backward wave reduces and oscillates in cycles, which is formed by reflections in the cardiovascular system. Therefore, the fluctuation of the backward wave can reflect the elastic performance of blood vessels. The backward wave obtained by the double triangular method is fitted well in the systole. Fiducial points from ejection duration can be obtained accurately. The time difference (T-reflection in Fig. 3) of the first and the second peaks of the backward wave can be calculated. The calculated time difference is used to estimate the cfPTT by a linear model which is obtained according to the training data from linear regression. The validation data is then used to test the model. The measurement of cfPTT from the carotid artery to the femoral artery is used as the gold standard. Then the aortic pressure wave is calculated by a transfer function, decomposed into forward wave and backward wave from the single triangular method^17^. The time difference between forward wave and backward wave of aortic pressure wave (TR_2_, as shown in Fig. 4) is calculated to estimate the cfPTT, similarly.

**Figure 5:**
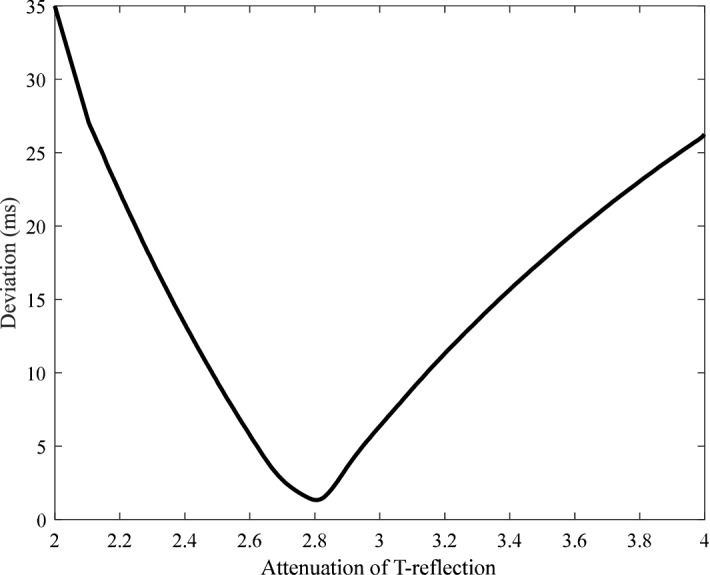
The variation of deviation with the attenuation ratio of T-reflection.

Because the start of backward wave cannot be determined, the distance traveled by the backward wave cannot be also calculated. In order to reduce the deviation between the T-reflection and cfPTT, an attenuation rate is added to the T-reflection, as shown in Fig. 5. In order to observe the influence of age on experimental data, the transit time ratio (TT_Ratio_) is introduced. The parameter is calculated according to Eq. (7). The lag is the T-reflection and TR_2_ in the radial artery and the aorta, respectively.

7 $$ {\text{TT}}_{\text{Ratio}} = {\text{lag}}/{\text{cfPTT}} $$

### Statistical analysis

Measured cfPTT and estimated PTT were analyzed by a paired t-test (IBM SPSS Statistics, version-23) and reported as mean ± SD or 95% CI where appropriate. The linear regression analysis and Pearson’s correlation coefficients were reported between measured cfPTT and estimated PTT. Bland–Altman plots were constructed to assess the agreement between estimated PTT versus measured cfPTT.

## Results

### Measured and estimated waves

The feature points were stable during the 5 min recording. The time of reflection, ejection time and the time of the dicrotic segment peak in pressure were 0.109 ± 0.01, 0.29 ± 0.04 and 0.51 ± 0.01 s. Therefore, the ratio of the time of reflection point to pressure wave was 10.30 ± 0.10%. The ratio of the time of the dicrotic segment peak to pressure wave was 47.90 ± 3.00%. The mean RMSE between the double triangular wave and the measured flow wave was 4.80 mmHg and the mean correlation was 0.87. As for the single triangular morphology, the mean RMSE with the measured flow wave was 7.30 mmHg and the mean correlation coefficient was 0.80.

**Figure 6:**
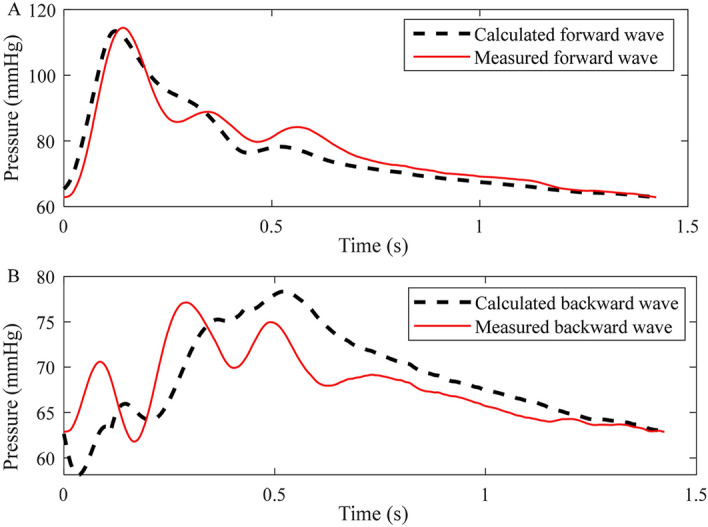
The measured and calculated waves of a representative subject. (**A**) Forward waves; (**B**) backward waves.

For the forward wave, the mean RMSE was 1.00 mmHg and the mean correlation coefficient was 0.98. For the backward wave, the mean RMSE was 1.00 mmHg and the mean correlation coefficient, 0.75. The comparison between the measured and calculated waves is shown in the Fig. 6. Through the verification of the measured flow wave, the double triangular flow wave can accurately approximate the measured flow wave. As for the single triangular method, between the ground truth and calculated decomposition, the mean RMSE was 1.70 mmHg and mean correlation coefficient was 0.95 for the forward waves. For the backward waves, the mean RMSE was 1.70 mmHg and the mean correlation coefficient was 0.69. Particularly, because the sum of forward and backward waves are pressure waves, the corresponding RMSE errors are always equal. The mean value of RM and RI was 0.86 and 0.46, respectively. The mean correlation coefficient between blood flow and double triangular flow was 0.88. The reflection point, start point of the second triangle and peak of the second triangle remained steady for the 5-min recording period.

### Measured cfPTT and estimated T-reflection

**Figure 7:**
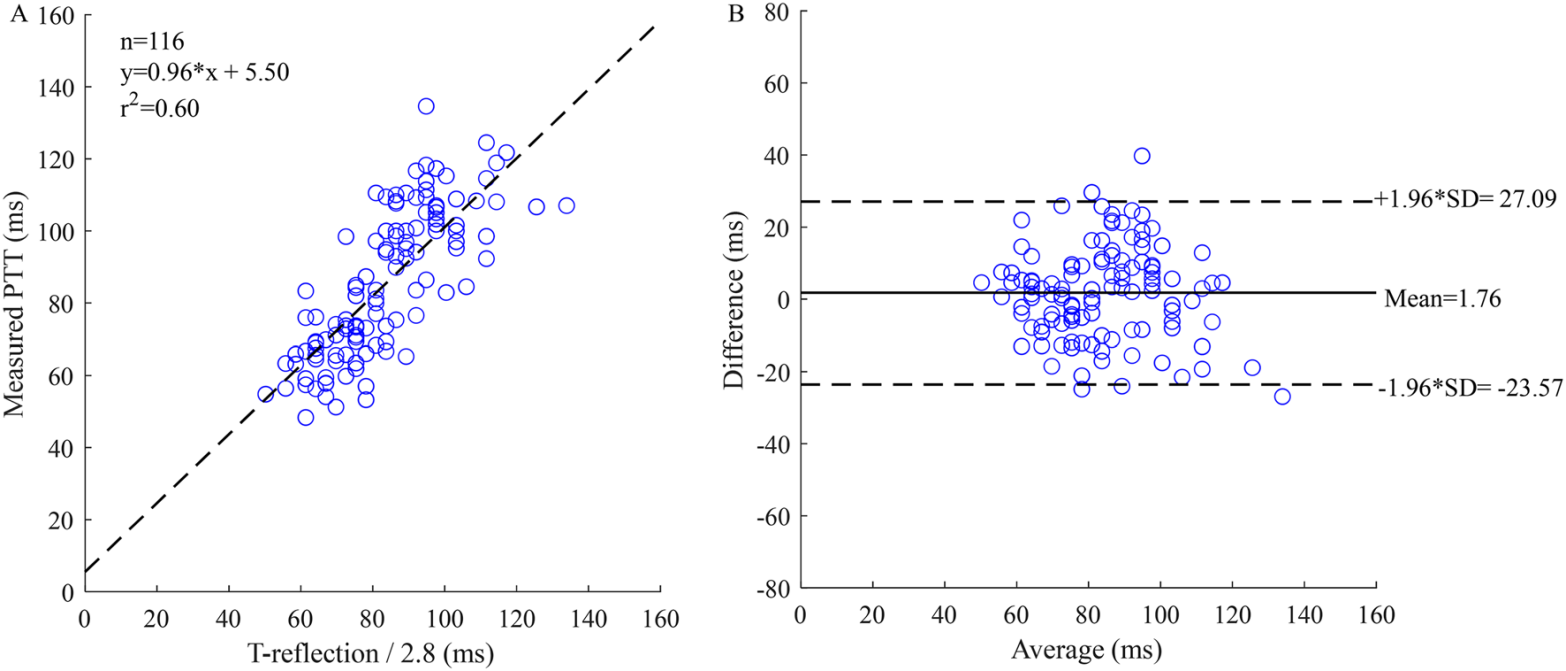
Correlation graphs and Bland–Altman plots comparing measured and calculated PTTs. (**A**) Scatter plot of the calculated PTT (T-reflection/2.8) vs measured carotid to femoral transit time (cfPTT) with regression line (dashed line) for all data. (**B**) Bland Altman plot of T-reflection/2.8 vs cfPTT for all data. Solid line is the mean difference, and the dashed lines are ± 1.96 SD of the difference. Difference: Measured cfPTT − calculated PTT; Average: (Measured cfPTT + calculated PTT)/2.

T-reflection can be calculated from the backward wave of decomposition. From the result, the T-reflection/2.8 gave the minimal deviation from the cfPTT. In 116 data sets, a linear regression model was obtained between the measured cfPTT (y, ms) and calculated T-reflection/2.8 (x, ms) as shown in Fig. 7A. The regression equation (r = 0.77, p < 0.001) is shown as Eq. (8). The attenuation ratio 2.8 was selected after the statistical analysis based on all the collected data.

8 $$ {\text{y}} = 0.{\text{96x}} + {\text{5}}.{\text{5}}0 $$

**Figure 8:**
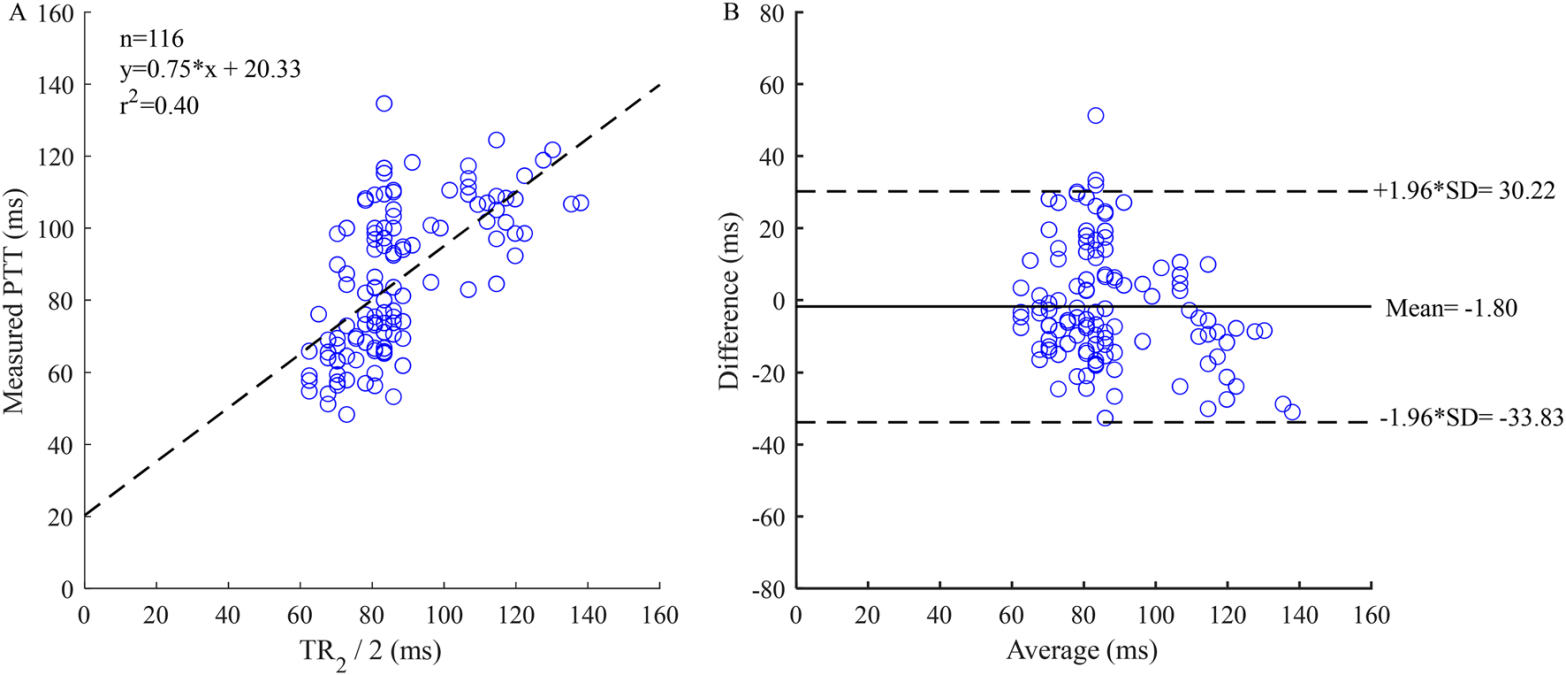
Correlation graphs and Bland–Altman plots comparing measured and calculated PTTs. (**A**) Scatter plot of the calculated PTT (TR_2_/2) vs measured carotid to femoral transit time (cfPTT) with regression line (dashed line) in all data. (**B**) Bland Altman plot of TR_2_/2 vs cfPTT in all data. Solid line is the mean difference, and the dashed lines are ± 1.96 SD of the difference. Difference: Measured cfPTT − calculated PTT; Average: (Measured cfPTT + calculated PTT)/2.

The results of measured cfPTT and calculated time delay based on the three different methods are listed in Table 2. The mean difference of the measured cfPTT and calculated T-reflection/2.8 was 1.76 ms, as shown in the Bland Altman plot (Fig. 7B). A linear regression model was obtained between the measured cfPTT (y, ms) and TR_2_/2 (x, ms; Fig. 8A). This regression equation is shown as Eq. (9). In addition, TR_2_ was calculated by analyzing the radial artery wave, but this parameter did not work well for the radial pulse wave decomposition. The correlation coefficient between the calculated TR_2_ of the radial pulse wave decomposition and measured cfPTT is 0.55. The mean difference of the measured PTT and calculated TR_2_/2 was 20.33 ms, as shown in the Bland Altman plot (Fig. 8B). A ten-fold cross-validation was used to verify the accuracy of the regression. The average of the cross validation was 0.64, and the standard deviation, 0.13. In the ten tests, the maximum value was 0.84 and the minimum was 0.49. The T-reflection and TR_2_ were substituted into Eq. (7) as the time lag, and the calculation results are shown in the Table 3. The two parameters showed the same trend with increase of age. From the calculation of TT_Ratio_ in different age groups, the values are increasing with age.

9 $$ {\text{y}} = 0.{\text{75x}} + {\text{2}}0.{\text{33}} $$

**Table 2:** Results of measured cfPTT and calculated time delay using different methods.

	Mean (ms)	SD (ms)
Measured cfPTT	85.68	20.37
Calculated T-reflection/2.8	83.92	16.49
Calculated TR_2_/2	87.49	17.31

**Table 3:** Age-related changes in transit time ratio.

Tertile	Age, mean ± SD	Range	Radial TT_Ratio_	Aortic TT_Ratio_
Group 1 (n = 41)	21 ± 3	18–25	1.33 ± 0.19	1.45 ± 0.27
Group 2 (n = 40)	42 ± 9	26–55	1.43 ± 0.23	1.57 ± 0.28
Group 3 (n = 35)	70 ± 9	56–92	1.45 ± 0.21	1.72 ± 0.25
Total (n = 116)	43 ± 21	18–92	1.40 ± 0.21	1.58 ± 0.29

In this study, according to the characteristics of radial artery blood flow, the decomposition of the aortic pulse wave was extended to a peripheral artery^23^. The transfer function is an important method for noninvasive data acquisition in the decomposition of the aortic pressure wave. However, the transfer function method is just an estimation of aortic pressure wave. Therefore, the extraction of feature points is not reliable, which is an important step of fitting the triangular flow wave. Compared with the aortic pressure wave, the radial pressure wave can be acquired directly and noninvasively. As listed in Table 4, the correlation between *V*_*e*_ and the time delay derived from the aortic pulse wave is positive. Here, *V*_*e*_ represents the elastic arterial PWV. If *V*_*e*_ increases, the aortic pulse transit time decreases. However, the time delay based on the decomposition of the aortic pulse wave is positively correlated with *V*_*e*_. Conversely, the time delay based on the decomposition of the radial pulse wave is negatively correlated with *V*_*e*_. The calculated results of the parameters (*V*_*e*_, *V*_*m*_, *D*_*e*_) for the aorta and radial artery are opposite, while a similar effect between the time lags and *D*_*m*_, HR, SV, R in aorta and radial artery is seen and is shown in Table 4.

**Table 4:** Correlation between parameters and time lags from different arteries.

Artery	*V*_*e*_	*V*_*m*_	*D*_*e*_	*D*_*m*_	HR	SV	R
Radial	− 0.43	0.32	− 0.41	0.13	0.32	0.05	0.03
Aorta	0.49	− 0.16	0.11	0.19	0.03	0.08	0.03

## Discussion

This study extends the wave reflection by decomposing the radial pressure wave and estimating the time lag of the fluctuation of the backward wave to calculate the cfPTT. The process of cfPTT estimation is a simple method based on the radial pressure wave alone. In order to explore the backward wave in the radial artery, a database of virtual healthy subjects’ pressure waves was used to decompose the aortic pressure wave to observe the influence of arterial parameters on the backward wave. The seven parameters are varying in different levels, which cause a change in the wave. As listed in the Table 3, both the time lags of aortic decomposition and radial backward wave are affected by the seven parameters. In particular, the SV and the R are changed every two or three sets and it is difficult to explore the relation among the time lags and the two parameters. The effects of aging on central elastic arteries and distal muscular arteries are opposing^32–34^, the correlations between *V*_*e*_, *D*_*e*_ and lag from the radial pressure wave are negative. It is obvious from the results that the time lag of the radial pressure wave can reflect more parameter changes. In contrast to the aortic lag, the radial lag may be applied in the future to arrhythmias and general examinations.

**Figure 9:**
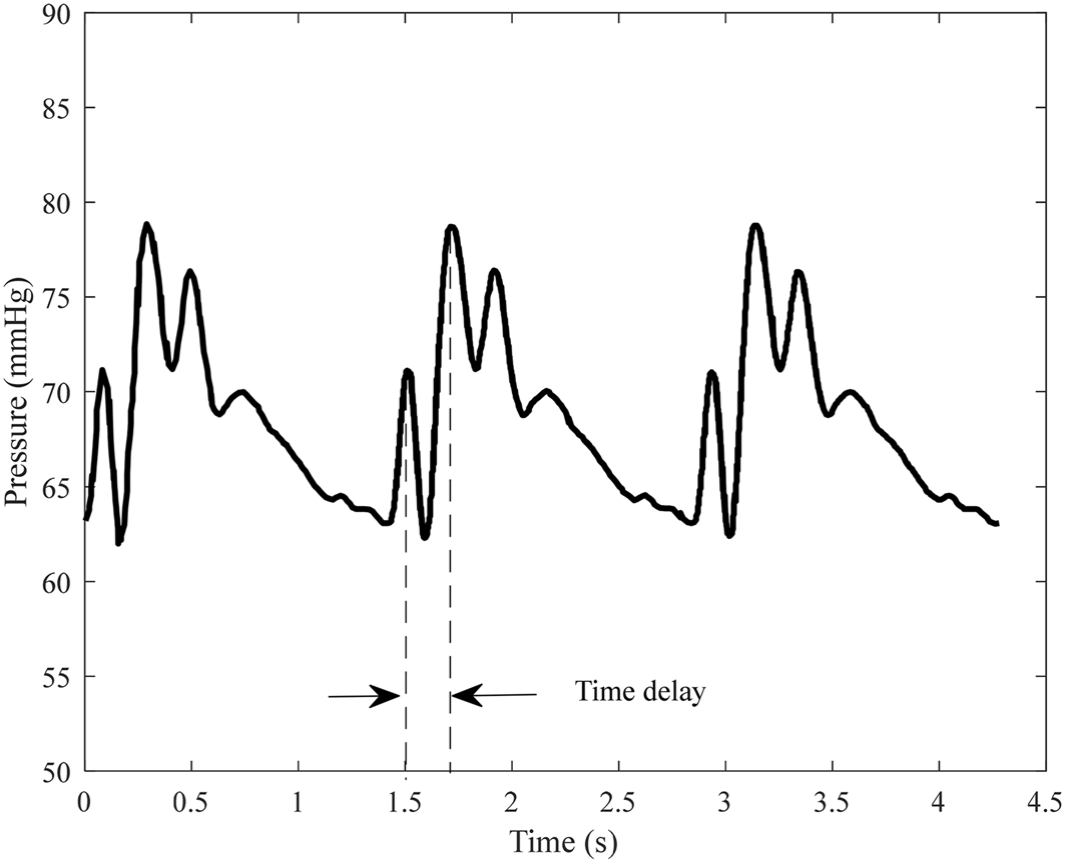
Backward wave calculated from the measured flow wave and pulse wave.

The verification has shown that the backward travelling component of the radial pressure wave can provide a noninvasive and nonintrusive estimation of cfPTT. The time lag of the fluctuations in the backward wave has a strong association with cfPTT. It is assumed that the fluctuations are caused by re-reflections in arteries^35^. The approach can be used for a noninvasively assessing aortic stiffness. However, the traditional cfPWV is the gold standard for noninvasive assessment of aortic stiffness. The carotid and femoral pressure waves are recorded simultaneously or determined with reference to the R wave of the ECG signal. The femoral pressure wave cannot be readily obtained when the patients are obese. Therefore, the proposed method can be used as a surrogate for the cfPTT. As shown in the Fig. 9, an example of a backward wave is calculated by the measured flow wave and pressure wave in the radial artery. Considering the reflection of a single period, the backward wave is causal with the pulse wave. Therefore, the fluctuation may represent the generation of a reflection. As seen in the backward wave, the fluctuations exist throughout the cycle. Because the diastole of ultrasonic blood flow has been unnoticed, the decomposition in diastole is not enough clear. In systole, peaks of fluctuation are distinguished.

From the point of view of the backward waves, the start point is different from the start point of the pressure wave. The reflection point represents the start of the backward wave; the true start of the wave occurs shortly before the reflection point. From the true starting point, the results show that the reflections are reduced and oscillated. This phenomenon confirms the existence of multiple pressure wave reflections. Therefore, reduction and oscillation of backward wave may be used to assess arterial stiffness.

The method of fitting blood flow to decompose a pressure wave has been studied. However, there are still drawbacks in the decomposition especially in diastole. Since the diastolic pulse wave contains less information and this method can be calculated from pulse wave itself, this convenient method has been widely used.

## Conclusions

From a single radial pressure wave, a new method to calculate the decomposition by an assumed double triangle flow wave has been proposed. From the backward wave, a time lag can be calculated from fluctuations which can be used to assess arterial stiffness. This result has been compared with aortic PTT calculated using the decomposition of the aortic pressure wave, cfPTT measured by an independent noninvasive method and the database based on a one-dimensional model. The time lag obtained from the radial pressure wave alone can serve as a surrogate measurement for cfPTT.

## Abbreviations

BSI: Blind system identification
CfPTT: Carotid-femoral pulse transit time
CfPWV: Carotid-femoral pulse wave velocity
*D*_*e*_: Elastic arterial diameter
*D*_*m*_: Muscular arterial diameter
HR: Heart rate
P_b_: Backward pressure wave
P_f_: Forward pressure wave
P_m_: Measured pressure wave
Q_b_: Backward pressure wave
Q_f_: Forward flow wave
Q_m_: Measured flow wave
R: Peripheral vascular resistance
RI: Reflection index
RM: Reflection magnitude
SV: Stroke volume
TF: Transfer function
*V*_*e*_: Elastic arterial PWV
*V*_*m*_: Muscular arterial PWV
